# The immune system in the control of microbiota homeostasis

**DOI:** 10.1186/1824-7288-41-S1-A29

**Published:** 2015-09-24

**Authors:** Giuseppe Penna, Maria Rescigno

**Affiliations:** 1 Department of Experimental Oncology, European Institute of Oncology, and Department of health Sciences, Universita’ di Milano, Via Adamello 16, Milan, Italy

## Background

In the intestine, dendritic cells (DCs) are found in the lamina propria (LP) of the villi, in the mesenteric lymph nodes (MLN), lymphoid aggregates and Peyer's Patches (PP). Probably the most represented antigen presenting cells in the gut are those found in the LP as they definitely outnumber the number of DCs found in the MLN or PP. In the mouse, these mononuclear phagocytes can be divided into subgroups depending on the expression of CX3CR1 (the receptor of fraktalkine) and CD103 ( E iIntegrin). CD103+ conventional DCs become tolerogenic in the gut, via their interaction with the local microenvironment and in particular with epithelial cells. Indeed, at steady state, ECs *(Epithelial cells)*condition anti-inflammatory DCs through the constitutive release of TSLP, TGF-b and retinoic acid (RA). EC-conditioned DCs even though phenotypically activated by bacteria polarize T cells towards a mucosal non-inflammatory T helper-2 phenotype or T regulatory cells. CX3CR1+ cells are instead apt at bacteria and food antigen uptake that they then transfer to CD103+ DCs via a gap junction-dependent mechanism. The interaction allows the establishment of tolerance to luminal antigens [1].

The microbiota can influence the cross-talk between immune cells and the mucosal microenvironment [2]. Thus the interplay between nutrition, microbiota and immune cells is decisive for the subsequent health of the infant. The rapid expansion of commercially available fermented food products raises important safety issues particularly when infant food is concerned. In many cases, the activity of the microorganisms used for fermentation as well as what will be the immunological outcome of fermented food intake is not known. 

## Materials and methods

We used established *in vivo*, *in vitro* and *ex vivo* models of infection/inflammation to study the immunomodulatory effects of fermented products of Lactobacillus paracasei CBA L74.

## Results

We found *in vitro* and *ex-vivo* that fermented products of Lactobacillus paracasei CBA L74 act via the inhibition of proinflammatory cytokine release leaving anti-inflammatory cytokines either unaffected or even increased in response to Salmonella typhimurium. These activities are not dependent on the inactivated bacteria but to metabolic products released during the fermentation process. Indeed CBA L74 fermented products (both culture medium and fermented milk) could protect against colitis and against an enteric pathogen infection (Salmonella typhimurium). Hence we found that fermented products can act via the inhibition of immune cell inflammation and can protect the host from pathobionts and enteric pathogens [3]. 

## Conclusions

These results open new perspectives in infant nutrition and suggest that L. paracasei CBA L74 fermented formula can provide immune benefits to an immature infant immune system.
